# Contribution of plant food bioactives in promoting health effects of plant foods: why look at interindividual variability?

**DOI:** 10.1007/s00394-019-02096-0

**Published:** 2019-10-22

**Authors:** Christine Morand, Francisco A. Tomás-Barberán

**Affiliations:** 1 Université Clermont Auvergne, INRA, Unité de Nutrition Humaine, UNH, CRNH Auvergne, Clermont-Ferrand, France; 2 Food and Health Laboratory, Research Group on Quality, Safety and Bioactivity of Plant Foods, CEBAS-CSIC, Campus de Espinardo, Murcia, Spain

**Keywords:** Human nutrition, Plant foods, Food phytochemicals, Cardiometabolic health, Interindividual variability, Personalised nutrition

## Abstract

**Purpose:**

Research has identified plant-based diets as the most protective for our health; it is now essential to focus on good food associations and the beneficial constituents in plant foods. From a growing body of evidence, some categories of food phytochemicals are increasingly considered to play a crucial role in the cardiometabolic health effects associated with plant food consumption. However, the heterogeneity in responsiveness to plant food bioactive intake that is frequently observed in clinical trials can hinder the identification of the effects of these compounds in specific subpopulations and likely lead to underestimating their actual contribution to the health effects of their food sources.

**Results:**

The magnitude and the main factors responsible for this between-subject variation in response to the consumption of the major families of food phytochemicals have been poorly documented so far. Thus, research efforts in this area must be developed. More importantly, capturing the interindividual variability in response to plant food bioactive intake, together with identifying the main determinants involved, is a crucial step that will enable the development and production of plant food products, thereby satisfying the nutritional needs and conferring benefits to different categories of populations.

**Conclusion:**

The development of a science-based personalised nutrition approach focusing on plant foods rich in specific bioactive compounds could contribute to alleviating the dramatic burden of metabolic and cardiovascular diseases.

## Key messages

- Plant food bioactives represent a tremendous potential for innovation in the field of food, nutrition, and health.
- The role of phytochemicals in the health benefits of plant-based foods has been underestimated so far.
- A full consideration of plant food bioactives in future preventive strategies implies research that tackles the factors responsible for interindividual variability.
- A better understanding of interindividual variability in response to plant food bioactives is needed to:
  - Refine dietary recommendations towards foods that are particularly rich in specific bioactives.
  - Broaden perspectives for the food industry to develop strategies for future personalised food products targeted to specific consumer groups.

## Introduction

A primary goal of nutrition research is to provide science-based knowledge to optimise health, preventing or delaying disease development. A growing body of evidence indicates that dietary interventions, especially those giving high intakes of plant foods, may delay or prevent the progression of age-related chronic diseases by protecting critical physiological functions of the body. Importantly, dietary patterns rich in plant foods have demonstrated to improve a diversity of intermediary markers of cardiometabolic risk, including blood pressure, glucose–insulin homeostasis, blood lipids and lipoproteins, endothelial function, inflammation, oxidative stress and possibly coagulation/thrombosis [1] and also to modulate the composition and functionality of the gut microbiota with a beneficial impact for human health [2]. Plant foods and many of their derived products (beverages, extracts) contain fibres and a variety of micronutrients (minerals, vitamins) and bioactive compounds, also called phytochemicals. The latter are secondary metabolites often produced by the plant in response to biotic or abiotic stresses, and they have gained growing notoriety over the last decade due to emerging evidence of their role in the pleiotropic pathways of effects of foods.

The aim of the present paper is (1) to provide a quick overview of the evidence accounting for the interest in plant food bioactives (PFB) for human health, especially for the prevention of cardiometabolic diseases and (2) to raise awareness on the relevance of in-depth exploration of interindividual variability (IIV) in response to the consumption of the main categories of these dietary compounds.

## Food phytochemicals

The most important categories of phytochemicals in our diets include (poly)phenols (flavonoid and non-flavonoid compounds), carotenoids (other than pro-vitamin A, such as lutein or lycopene), sulphur compounds (glucosinolates and allyl sulphides), purine alkaloids (caffeine, theobromine) and plant sterols (phytosterols and phytostanols). The primary dietary sources of phytochemicals are beverages (coffee, tea, wine, beer, juices), fruits, vegetables, legumes, nuts, and grains. The majority of the phytochemicals are present in many different foods, while others are rather specific, as is the case of glucosinolates that are mainly present in vegetables of the Brassicaceae family (broccoli, kale, cabbage, radish), the allyl sulphides in members of the Alliaceae (onion, garlic, leeks), and the purine alkaloids occurring in non-alcoholic beverages (coffee, tea, mate and cocoa). The daily intake of phytochemicals can vary largely depending on the dietary habits of the individual. For (poly)phenols, the largest family of phytochemicals in the human diet, the intake can be as high as 2 g per day in some individuals [3].

The bioavailability of the (poly)phenols present in foods is often very low, and seldom reach values above 5% of the intake [4]. Thus, most of the dietary (poly)phenols reach the colon unmodified, where they interact with the commensal microbiota, being catabolized to provide relevant metabolites that are better absorbed and that show consistent biological effects [5]. Other phytochemicals, such as the purine alkaloids, are easily absorbed in the small intestine, and are extensively metabolised in the liver, while glucosinolates and allyl sulphides are hydrolysed by enzymes present in the raw food to release isothiocyanates, thiocyanates, and nitriles in the former, and allicin and related compounds in the case of alliin. In both cases, they can also be metabolised in the gut by interaction with the gut microbes [6]. The digestion of fat-soluble phytochemicals, such as carotenoids and plant sterols, occurs in the upper part of the gastrointestinal tract and depends on several successive steps involving several transporters and metabolising enzymes. The digestion process of these compounds begins with their extraction from food or supplements and incorporation into mixed micelles, followed by their uptake by the enterocytes, and lastly, the non-effluxed fraction is mostly secreted in the lymph fluid into chylomicrons, either as free or esterified molecules [7].

## Plant food bioactives and cardiometabolic health

Some of these plant food bioactives hold promise in reducing the risk of cardiometabolic disease and maintaining body functions as evidenced by a growing number of studies that provide convincing epidemiological and clinical data, strengthened by mechanistic insights from experimental studies. The more substantiated evidence in the field is primarily concerned with compounds from the polyphenol and phytosterol families. Several supplementation studies carried out in animal models support an interest in a diversity of (poly)phenols to protect cardiometabolic health, notably by attenuating atherosclerosis development, improving vasodilation, preventing diet-induced obesity, improving lipid profile and glucose metabolism and by displaying antioxidant and anti-inflammatory effects [8]. Although (poly)phenolic compounds generally exhibit a low bioavailability, results from mechanistic studies suggest that they can act in vivo as signal molecules. They affect gene expression and signalling pathways, exert epigenetic regulations, modulate enzyme activities, and interact with cell receptors and with the gut microbiota to ultimately regulate a number of critical biological and physiological processes involved in the control of vascular and metabolic health (e.g., nitric oxide bioavailability and bioactivity, cellular redox homeostasis, inflammatory status, insulin sensitivity, lipoprotein function, etc.) [9–11]. The most recent cell studies which have been carried out using omics approaches and with the plasma metabolites produced in vivo from food bioactive compounds have confirmed the capacity of these metabolites to display biological properties even at the low concentrations achievable in the context of a human diet [12, 13].

Recent meta-analyses of extensive prospective cohort studies have reported that the higher intakes of flavonoids were associated with a decreased risk (at least − 10%) of both Type 2 diabetes and cardiovascular diseases [14, 15]. (Poly)phenols are highly present in the essential foods of the MedDiet (extra-virgin olive oil, nuts, red wine, legumes, vegetables, fruits, and whole-grain cereals). From the PREDIMED study, (poly)phenols have been identified as key contributors to the beneficial effects of the MedDiet related to their ability to reduce blood pressure, improve glucose homeostasis and blood lipid profile and improve the anti-inflammatory and antioxidant status [16]. Many randomised controlled trials published over the last 15 years have reported on the beneficial effects of the acute or chronic intake of some (poly)phenols or (poly)phenol-rich foods on surrogate risk biomarkers of cardiovascular and metabolic diseases (blood pressure, endothelial function, blood lipids, fasting glucose, insulin, HOMA-IR), and thereby substantiated their protective role in cardiometabolic disease development [17–20].

Plant sterols/stanols inhibit the intestinal absorption of dietary cholesterol, and their LDL cholesterol-lowering effect has been reported in several meta-analyses of a vast number of randomised placebo control trials showing a dose–response relationship with intakes of 1.5–3 g/day lowering LDL cholesterol by 7.5–12% [21]. Because these effective daily doses cannot be obtained from natural sources with habitual diets (200–400 mg/d) [22], these phytochemicals are added to supplements or foods, most often incorporated into fat-containing foods. The LDL cholesterol-lowering effect of plant stanols/sterols is due to a reduced intestinal cholesterol absorption through competition and also to their impact on biological processes increasing the clearance of circulating LDL [23].

Some observational and experimental studies suggest that carotenoids can positively affect human health, mainly by protecting against ocular diseases, certain cancers and cardiovascular diseases [24]. As recently reviewed, through varied mechanisms, these compounds play a multifaceted role in the control of cellular redox homeostasis, with a potential impact on physiological processes and human health [25]. Among the carotenoids, lycopene has been reported to have a heightened antioxidant efficiency, and it is also the compound for which some intervention-based studies have reported to have protective effects on different components of metabolic syndrome, from a lycopene-rich beverage, despite varying doses and duration of intakes [26]. However, clinical evidence of the cardiometabolic health benefit of carotenoids is still scarce.

To date, only a few of these food phytochemicals have already been integrated into health claims by the EFSA panel. The approved health claims are related to cocoa flavanols and the maintenance of normal endothelium vasodilation [27], to the phenolics of olive oil for maintaining normal HDL cholesterol levels [28] and to the plant sterol-enriched foods for their cholesterol-lowering effects [29].

## Interindividual variability in response to consumption of plant food bioactives

Despite the accumulative scientific evidence supporting the interest in eating plant food bioactives to protect essential body functions and reduce the risk of developing cardiometabolic diseases, the human intervention studies aiming to demonstrate the effects of specific phytochemicals, or foods rich in, on intermediate biomarkers of cardiometabolic risk have often shown mixed results [30]. One major cause is the heterogeneity of the individual response to their intake, which reduced the significance of the effects on some biomarkers of health at the scale of the population studied, although their intake showed promising results in subgroups of subjects participating in the trials [31]. This between-subject variation in the response suggests that the consumption of particular foods or bioactive compounds may benefit some individuals more than others. However, up to now in published controlled trials, the IIV has been most often masked by the statistical analysis which produces the mean data of the study population rather than the individual’s data [32]. To overcome this issue, in future human trials, the data obtained for each subject should be published as those provide very informative data on the variation of the effect among the individuals. If the human trial is complemented with a study of the variation on food bioactive compound ADME, and with a measurement of the exposure to specific bioactive metabolites, a correlation between the biological effect on cardiometabolic health biomarkers, and the exposure to the bioactive metabolites could be evaluated. This approach will likely explain in large part the differences observed between responders and non-responders to the intervention and will allow identifying the metabolites responsible for the bioactivity in the body of the different categories of plant food bioactives. This heterogeneity in responsiveness has often led to inconclusive results in clinical trials examining the health effects of specific phytochemicals over the last decades, with the unfortunate underestimation of the actual role of PBF in the health benefits of plant-based foods being a possible consequence. A full consideration of PFB in the future strategy of personalised nutrition cannot escape from a first in-depth investigation of the factors responsible for the IIV in response to consumption of the major categories of PFB of our diet.

A range of factors such as genetic background, gut microbiota composition, pathophysiological status, age, or gender could explain these interindividual variations, and they may differ depending on the bioactive compounds. For example, the IIV in the bioavailability of carotenoids has been shown to depend on single nucleotide polymorphisms (SNPs) in genes involved in the intestinal uptake or efflux of these compounds as well as in genes involved in their metabolism and transport [7]. The phenotypic effect of each SNP is usually low, but combinations of SNPs can explain a significant part of the variability with impact on the carotenoid status of individuals. Variability in polyphenols ADME can also partly originate from differences in gut microbiota composition which determine the conversion or not of some categories of (poly)phenols into metabolites displaying higher bioactivity than the parent compounds and with potential impact on their health effects [33]. For example, different (poly)phenol metabotypes (reflecting the metabolic capacity of the gut microbiota towards dietary (poly)phenols) have been described for the gut microbial metabolism of soy isoflavones and ellagitannins from berries and nuts, depending on their conversion or not into equol and urolithins respectively [34, 35]. Clustering of individuals according to equol or urolithin metabotypes is consistent with the IIV observed in the improvement of cardiometabolic biomarkers in human intervention studies using isoflavones or ellagitannins [36, 37]. However, between-subject variations in the biological response to (poly)phenol consumption are not always linked to differences in bioavailability. For example, as observed in a parallel-group intervention study, at comparable levels of bioavailability, the lowering effect of cocoa flavanols on systolic blood pressure reached significance in young but not in elderly subjects [38]. Acute intake of curcumin has also shown to improve endothelial function in women only and not in men, whereas the plasma concentrations of curcumin were unchanged between the males and females enrolled in the trial [39]. Substantial heterogeneity in the individual LDL cholesterol response to phytosterol therapy has also been repeatedly reported in human interventions [40], and the variability has been mainly explained by polymorphisms across genes associated with cholesterol trafficking pathways [41]. Despite these few known examples, the factors responsible for variability in ADME and biological responsiveness for the main categories of PFB are far from being fully identified. The available studies do not allow to determine to what extent the between-subject variation in the ADME of PFB can explain the IIV in their biological responsiveness due to the lack of clinical trials investigating both the bioavailability and the biological effects [32].

Although some examples in the literature clearly illustrate that IIV in response to PFB consumption exists, only limited and scattered data are available on the subject. To tackle the complexity of this question through a multidimensional approach, the European COST POSITIVe network (https://www6.inra.fr/cost-positive) has gathered a large community of experts in nutrition and plant food bioactives, food science, clinical research, microbiology, gut microbiome, genetics, nutrigenomics, bioinformatics, cellular and molecular biology. These experts have joined their efforts to analyse systematically the currently fragmented knowledge in the field. The activities of this network aimed to (1) evaluate the extent of the IIV for the major categories of PFB and identify the main factors responsible for between-subject variation in both the ADME and the biological responsiveness regarding cardiometabolic endpoints; (2) integrate the main findings of the network to identify the gaps in knowledge and needs for future research and also to examine how this knowledge translates into concrete applications for the different categories of stakeholders; (3) provide some recommendations to better capture interindividual variation in intervention trials. The main results of the POSITIVe network are presented and discussed in the reviews of the special issue of the European Journal of Nutrition on interindividual variability in response to PFB.

## The knowledge of interindividual variability determinants as a tool for personalised nutrition

The studies of the COST Action POSITIVe have shown the complexity of the different determinants that govern the effects of PFB on cardiometabolic health. The activities of the POSITIVe network showed that many different factors could modulate the health effects of foods, and they should be considered when forecasting the health effects of a given phytochemical in one subject or population sub-groups. All of these determinants could be used when designing dietary interventions for personalised nutrition or precision nutrition.

The question is: how can this be implemented? First, the contribution of the different determinants to the health effects produced by food phytochemicals should be fully demonstrated and validated. Then the specific conditions of a given individual should be evaluated. Thus, analyses of gene variants related to nutrition and metabolism, and of gut microbiota composition and function should be completed. Evaluation of the nutritional status of the individual as well as disease story, anthropometric data and a range of other individual characteristics (e.g., gender, age, ethnicity) have to be considered as well. With these data, the potential health effects could be envisaged, and the more efficient dietary interventions or dietary habits recommendations could be established. The dose of the bioactive phytochemical to be administered could also be identified, although this is not an easy task, as many related phytochemicals can also exert effects and lead to synergies that are difficult to estimate with the current knowledge.

When this information is available and validated, mathematical models to show the best nutritional interventions for a given volunteer or population sub-groups to respond to the treatment with PFB will be developed.

Once the active phytochemicals are identified, and as their content in different food products is available in free online databases [Phenol explorer (http://phenol-explorer.eu/); Phytohub (http://phytohub.eu/)], various food products containing the specific phytochemical bioactives that produce the desired effects for a given subject or particular population group will be selected and specifically recommended.

Finally, there will be a need to develop consumer-affordable (simple, reliable, cost-effective) applications for self-classification and self-monitoring. These applications could also suggest some food associations and culinary preparations leading to optimal nutrition with a correct administration of the bioactive phytochemicals.

## Conclusion

Instead of continuing with the promotion of recommendations for fruits and vegetables to the population in a “one-size-fits-all” approach, without proving its efficiency during the last decades, it could be of special relevance to provide the scientific basis to evolve towards the elaboration of refined dietary recommendations that would ensure that everyone is adequately exposed to the protective constituents provided by these foods. Reaching this aim implies first gaining a good understanding of the interindividual variability and to be able to predict the individual response to PFB intake. Good knowledge of IIV will also broaden perspectives for the food industry by underpinning the development of new functional or optimised traditional foods with more pronounced health benefits for targeted consumer groups. Achieving these scientific developments will undeniably require substantial research efforts and funding.

## Abbreviations

ADME: Absorption–distribution–metabolism–excretion
COST: European Cooperation in Science and Technology
EFSA: European Food Safety Authority
HOMA-IR: Homeostasis model assessment of insulin resistance
IIV: Interindividual variability
LDL: Low-density lipoprotein
MedDiet: Mediterranean diet
PFB: Plant food bioactives
POSITIVe: Interindividual variability in cardiometabolic response to consumption of plant food bioactives
PREDIMED: Prevencion con dieta mediterranea
SNP: Single nucleotide polymorphism
